# Rudi Kundini, Pamoja Kundini (RKPK): study protocol for a hybrid type 1 randomized effectiveness-implementation trial using data science and economic incentive strategies to strengthen the continuity of care among people living with HIV in Tanzania

**DOI:** 10.21203/rs.3.rs-3315136/v1

**Published:** 2023-12-20

**Authors:** Jillian L. Kadota, Laura J. Packel, Matilda Mlowe, Nzovu Ulenga, Natalino Mwenda, Prosper F. Njau, William H. Dow, Jingshen Wang, Amon Sabasaba, Sandra I. McCoy

**Affiliations:** University of California Berkeley School of Public Health; University of California Berkeley School of Public Health; Health for a Prosperous Nation; Management and Development for Health; Rasello; Ministry of Health; University of California Berkeley School of Public Health; University of California Berkeley School of Public Health; Health for a Prosperous Nation; University of California Berkeley School of Public Health

**Keywords:** Effectiveness-implementation hybrid, HIV/AIDS, conditional cash transfers, economic incentives, Tanzania

## Abstract

**Background:**

Economic incentives can improve clinical outcomes among in-care people living with HIV (PLHIV), but evidence is limited for their effectiveness among out-of-care PLHIV or those at-risk of disengagement. We propose a type 1 hybrid effectiveness-implementation study to advance global knowledge about the use of economic incentives to strengthen the continuity of HIV care and accelerate global goals for HIV epidemic control.

**Methods:**

The Rudi Kundini, Pamoja Kundini study will evaluate two implementation models of an economic incentive strategy for supporting two groups of PLHIV in Tanzania. Phase 1 of the study consists of a two-arm, cluster randomized trial across 32 health facilities to assess the effectiveness of a home visit plus one-time economic incentive on the proportion of out-of-care PLHIV with viral load suppression (<1000 copies/ml) 6 months after enrollment (n = 640). Phase 2 is an individual 1:1 randomized controlled trial designed to determine the effectiveness of a short-term counseling and economic incentive program offered to in-care PLHIV who are predicted through machine learning to be at-risk of disengaging from care on the outcome of viral load suppression at 12 months (n = 692). The program includes up to three incentives conditional upon visit attendance coupled with adapted counselling sessions for this population of PLHIV. Consistent with a hybrid effectiveness-implementation study design, phase 3 is a mixed methods evaluation to explore barriers and facilitators to strategy implementation in phases 1 and 2. Results will be used to guide optimization and scale-up of the incentive strategies, if effective, to the larger population of Tanzanian PLHIV who struggle with continuity of HIV care.

**Discussion:**

Innovative strategies that recognize the dynamic process of lifelong retention in HIV care are urgently needed. Strategies such as conditional economic incentives are a simple and effective method for improving many health outcomes, including those on the HIV continuum. If coupled with other supportive services such as home visits (phase 1) or with tailored counselling (phase 2), economic incentives have the potential to strengthen engagement among the subpopulation of PLHIV who struggle with retention in care and could help to close the gap towards reaching global ‘95-95-95’ goals for ending the AIDS epidemic.

Phase 1: Clinicaltrials.gov, NCT05248100, registered 2/21/2022 https://clinicaltrials.gov/ct2/show/NCT05248100
Phase 2: Clinicaltrials.gov, NCT05373095, registered 5/13/2022 https://clinicaltrials.gov/ct2/show/NCT05373095

## BACKGROUND

Antiretroviral therapy (ART) has been scaled up rapidly over the last 15 years, with over 28 million people living with HIV (PLHIV) accessing ART in 2021, compared to 7.8 million in 2010 (1). The benefits of early ART are well-documented (2, 3), with sustained treatment adherence resulting in reductions in morbidity and mortality, as well as the prevention of onward transmission of the virus (4). However, even with expanded access to ART and HIV care through ‘Treat All’ initiatives (3), reaching the Joint United Nations Programme on HIV/AIDS (UNAIDS) ‘95-95-95’ goals for ending the AIDS epidemic by 2030 will be challenging (5). In eastern and southern Africa, which accounts for 55% of HIV infections globally, only 72% of adult PLHIV have viral suppression (6), a partial reflection of poor linkage to care, suboptimal adherence, and persistent disengagement from care that undermines the goals of “treatment as prevention” (TaSP) programs. A large study conducted across 22 countries in sub-Saharan Africa in 2018 revealed that 5 years following treatment initiation, the cumulative incidence of PLHIV who were either lost to follow up (LTFU) or had stopped ART was 19%, while 15% had died (7). These findings demonstrate a critical need for new strategies to improve retention in care among PLHIV, especially strategies designed to address the dynamic nature of lifelong engagement in care.

While “Treat All” removed key barriers that prevented early ART initiation, expanded access to ART and HIV care services, and became the new paradigm that harnesses the benefits of TaSP there remain challenges among PLHIV in accessing and engaging in continual HIV care and treatment. For many PLHIV in eastern and southern Africa, the pathway to lifelong ART success including retention in care is hampered by stigma, high levels of food insecurity, negative clinic experiences, anticipated or actual side effects, misinformation, asymptomatic infection, “treatment fatigue”, and myriad factors related to poverty (8–15). Consequently, retention can be a dynamic process as some PLHIV may disengage and re-engage in care numerous times over a lifetime (14, 16, 17). Furthermore, it is increasingly recognized that PLHIV are heterogeneous in their ability to mitigate barriers. Some groups need minimal support to be successful, while others continually struggle to maintain continuity of care and/or viral suppression (18–21). This subset of out-of-care PLHIV and/or those with detectable viremia can play an outsized role in onward transmission; thus, innovative strategies focused on these PLHIV is central to epidemic control. Indeed, robust approaches for linkage and retention are a critical research priority for sub-Saharan Africa in the era of universal treatment (22).

Short-term, economic incentives are a proven strategy to improve adherence and retention among PLHIV starting ART (23, 24). This is based on a substantial foundation of existing evidence suggesting that by partly mitigating structural barriers like poverty and food insecurity and overcoming motivational roadblocks to care (10, 12, 13, 25, 26), incentives can increase retention, ART adherence, and viral suppression among ART initiates and in-care PLHIV (26–33). However, there is little empirical evidence demonstrating their effectiveness in improving outcomes including re-engagement for out-of-care or at-risk PLHIV, despite a strong theoretical rationale (23). Specifically, it is increasingly recognized that disengagement from care is a process beginning with everyday competing demands that results in missed visits, which slowly evolves into a reluctance to return and the erosion of connection to care (14).

Per behavioral economics, Self-Determination Theory (SDT), and microeconomic theory, incentives may overcome this reluctance including by mitigating small immediate costs in order to nudge PLHIV to engage or re-engage with care, and/or by decreasing the cost of visit attendance or adherence, which in turn increases demand (34–36).

Recognizing this gap, in 2018 we co-designed an intervention consisting of a small, scalable, one-time incentive (~$10 US Dollars (USD)) coupled with an existing system of home-based care (HBC) providers who locate PLHIV disengaged from care. This leveraged learnings from a multi-year process co-designing and evaluating monetary and non-monetary incentive programs for PLHIV with local research partners, PLHIV, and Ministry of Health stakeholders (33, 37, 38). Consistent with President’s Emergency Plan for AIDS Relief (PEPFAR) guidelines, disengagement from care was defined as not attending a clinic appointment for ≥ 28 days since the last scheduled appointment (39–41). In a 2-armed randomized controlled pilot study of 157 out-of-care PLHIV in Tanzania, we found that our intervention was feasible, acceptable, and demonstrated early signals of motivating re-engagement: 86% returned within 3 months in the intervention group vs. 78% in outreach/referral only (adjusted risk difference (RD) = 0.08, 95% Confidence Interval (CI): −0.03, 0.19) (42). We also found the intervention did no long-term harm: among those who linked to care, there were a similar number of completed *non-incentivized* visits in the 6 months after the intervention period was complete in the incentive and comparison groups (median 2 visits), suggesting that PLHIV in the incentive group attend future, non-incentivized visits at high levels, similar to their non-incentivized peers.

These promising results suggest that the current type 1 hybrid effectiveness-implementation study (43) is now warranted, along with expansion to reach PLHIV who are in care but at risk of disengaging from care. Our central hypothesis is that the economic incentive intervention will motivate PLHIV to re-engage or to stay engaged in HIV care and adhere to ART, thereby increasing the proportion of PLHIV with viral suppression and thus leveraging the full potential of TasP moving us closer to the ‘95-95-95’ goals. In Rudi Kundini, Pamoja Kundini (RKPK, *Return to Care, Together in Care* in Kiswahili), we will use a three-phased approach to assess the impact of this economic incentive strategy using different implementation models tailored to two populations of PLHIV – those out-of-care and those predicted to be at-risk of disengagement. At the project’s end, we will understand the effectiveness of these intervention strategies for two vulnerable groups of PLHIV and implementation factors that drive impact, consistent with an implementation science approach to close the gap between evidence and practice (44, 45).

## METHODS/DESIGN

### Design

**Figure 1:**
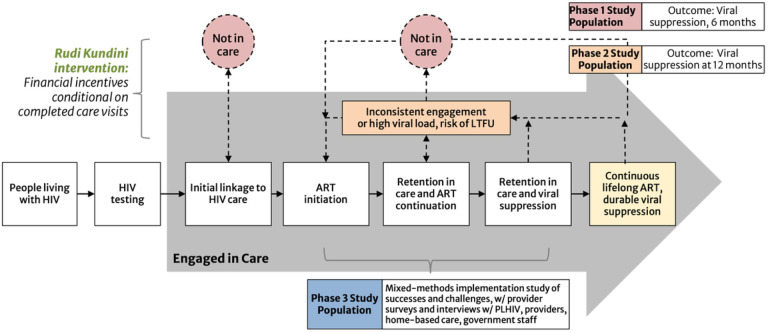
Proposed Impact Pathway. Integration of incentives into the HIV care continuum and populations for each phase. Boxes in the grey arrow show the steps of care, including lifelong antiretroviral therapy. The dashed lines, orange box, and pink circles display the process of defaulting and re-engaging in care. In the proposed project, incentives will be used as part of the Rudi Kundini, Pamoja Kundini intervention to encourage engagement in care among people who: 1) have disengaged from care (pink circles), and 2) people at risk of LTFU (orange box). ART=antiretroviral therapy; LTFU=loss to follow-up.

The overall objective of RKPK is to evaluate two implementation strategies of an economic incentive intervention for supporting PLHIV struggling with continuity of care in Tanzania (Fig. 1). The study includes three distinct phases:

1. Phase 1 is a cluster randomized trial of PLHIV disengaged from HIV care in which 32 health facilities will be randomized in a 1:1 ratio to standard of care (SOC) or the economic incentive intervention. The pre-registered primary endpoint (ClinicalTrials: NCT05248100) is viral load suppression at 6 months, defined as the proportion of PLHIV on ART and with suppressed HIV viral load (< 1000 copies/ml) 6 months after enrollment. The intervention implementation model is a home visit by an HBC provider (SOC in Tanzania mandates that PLHIV who are identified as LTFU from an HIV clinic or who miss a regularly scheduled appointment are initially be traced and contacted by an HBC provider), plus a one-time incentive for returning to care.
2. Phase 2 is a 2-armed, parallel 1:1 individually randomized controlled trial conducted at two high volume health facilities to evaluate the effectiveness of a short-term economic incentive program offered to in-care PLHIV who are predicted to be at-risk of disengaging from HIV care using a machine learning algorithm developed using routinely collected medical and pharmacy electronic medical record (EMR) data from the parent regions. The pre-registered primary outcome is the proportion of PLHIV on ART and with suppressed HIV viral load (< 1000 copies/ml) 12 months after enrollment (ClinicalTrials: NCT05373095). The intervention implementation strategy includes up to 3 monthly incentives conditional upon visit attendance and attendance at “Pamoja Kundini” counseling (PKC) sessions, adapted from SOC enhanced adherence counselling (EAC) sessions designed to address the challenges of long-term HIV care engagement with respect and empathy.
3. Phase 3 will explore implementation challenges and successes using a mixed methods design. We will conduct surveys and in-depth interviews (IDIs) to assess barriers and facilitators to phase 1 and 2 implementation for both groups of PLHIV and from multiple stakeholder perspectives including health facility staff, HBCs, and government staff. Guided by the Consolidated Framework for Implementation Research (CFIR) (46) we will examine individual (perceptions, motivations), intervention (barriers, facilitators), and contextual factors (management, policies) that influence intervention effectiveness.

The protocol is registered on Clinicaltrials.gov and complies with SPIRIT reporting guidelines (Additional File 1). The study includes an independent Data and Safety Monitoring Board that provides oversight of patient safety, adverse events, any study changes, and study data quality/integrity.

### Implementation Science Frameworks and Theoretical Models Guiding Intervention Design

#### Hybrid Effectiveness-Implementation trial designs

Hybrid effectiveness-implementation trials are designed to accelerate translation of evidence-based findings to routine practice by blending clinical effectiveness research with implementation science methods (43). These studies can range in classification from type I to III; on this spectrum, the RKPK study is type I: we primarily aim to determine the effectiveness of two types of economic incentive strategies on study outcomes (phases 1 and 2), with a secondary aim of understanding implementation successes/challenges (phase 3).

#### Psychology and Economic Theories Underlying the Intervention

The use of economic incentive strategies for two populations of PLHIV in this study is supported by several economic and psychological theories. In phase 1, we will examine the effect of a one-time “nudge” (35) to reconnect out-of-care PLHIV to care (47, 48). When a behavior, like re-linkage to care, has small immediate costs and large delayed benefits, a small immediate incentive may counteract present costs and tip the balance towards the positive behavior. This is also predicted by SDT, which describes engagement in an activity because of an external reward like an incentive (36). In phase 2, the desired behavior – retention in care among PLHIV who are at-risk of disengagement – is complex, necessitating more than a one-time nudge (49). Per microeconomic theory, incentives for retention in care decrease the cost of visit attendance or adherence, which in turn increases demand (34). Although many PLHIV struggling with continuity of care face short-term structural barriers to care, they may also have insufficient habit formation and/or other behavioral barriers (e.g., mental health conditions). This co-occurrence of behavioral and structural hurdles justifies the longer proposed incentive period of three months to reinforce habits (50, 51), plus incentive delivery alongside supportive, educational, monthly PKC.

#### Health facility eligibility and recruitment

For phase 1, we will work with the Regional Medical Officers (RMOs) in Geita and Kagera Regions (Lake Zone) to generate a list of HIV care facilities currently using an EMR database and that had at least 750 PLHIV on ART in any quarter of 2021. We will proceed with a two-part facility selection process: separately by region, we will use ArcGIS geographic information system software to randomly select a set of up to 25 facilities ≥ 15 kilometers from any other facility in the list. From this list, we will randomly select 16 facilities per region for study inclusion. Phase 2 includes two purposively selected large facilities in Geita Region.

#### Participant eligibility and recruitment

##### Phase 1 – *Out-of-care PLHIV*:

At participating health facilities lists of former clients who are classified as LTFU or having missed recent appointments will be generated from EMR data, typically quarterly, and distributed to HBC providers who trace PLHIV at their provided home address. We will work within this existing system to recruit phase 1 participants. After completing standard procedures, HBCs will assess potential participants for study eligibility criteria including: 1) living in the catchment area of a study health facility; 2) ≥ 18 years; 3) ownership of a phone/has consistent phone access; 3) classified as LTFU from HIV care or missing appointments (not attended a clinic appointment for ≥ 28 days since last scheduled appointment), and 4) has had a clinic appointment within the last 24 months. HBCs will obtain written informed consent among those eligible and interested in participation.

##### Phase 2 – *At-risk, in care PLHIV*:

Eligible PLHIV are those: 1) currently on ART and with a valid viral load result in the last 6 months (indicating current or recent care engagement); 2) not currently enrolled EAC in nor starting EAC sessions within one week; 3) ≥ 18 years; 4) with ownership of a phone/with consistent phone access; 5) living in Geita Region and intending to receive care at a study facility for the next 12 months, and 6) classified as “high-risk” for disengagement from HIV care using our machine learning algorithm. We will consider for inclusion in the algorithm routinely collected medical and pharmacy EMR data that partially explain observed information about viral suppression and/or retention, including patterns of viral load, adherence, and visit attendance. We will then develop a predictive model using data from the participating regions to identify PLHIV who are at high-risk for disengaging from care, having high viral load, or death. Using a facility-generated dataset of clients currently accessing care at the facility, our study team will create a list of potentially “high-risk” clients via our machine learning model, which will then be used by health facility staff to recruit and enroll clients for study participation.

##### Phase 3

We will recruit and invite a subset of PLHIV who participated in phase 1 or 2 for participation in IDIs and will purposively select health facility staff and HBCs who participated in study implementation for participation in surveys and IDIs (Table 1). Consistent with our goal to optimize intervention strategies for wider scale-up, if found effective, we will recruit key government stakeholders identified as having authority over health-related activities in study communities for participation in IDIs.

#### Study arms

##### Phase 1 – *Out-of-care PLHIV*:

Participants living in catchment areas of facilities randomized to the comparison arm will receive SOC services, which according to Tanzania’s National Guidelines for the Management of HIV and the Ministry of Health includes: 1) HBC tracing of PLHIV who have disengaged from primary care, 2) provision of counseling to return to HIV care, and 3) an offer to schedule an HIV primary care appointment on the spot. Participants living in intervention facility catchment areas will receive the same SOC HIV tracing and clinical services as comparison participants, plus the opportunity to receive a one-time incentive of 22,500 Tanzanian Shillings (TSH), with half (11,250 TSH) delivered via mobile money upon enrollment and half delivered after confirmation of a completed clinical visit if within 90 days of study enrollment.

##### Phase 2 – At-risk, in care PLHIV

Participants randomized to the comparison arm will receive SOC HIV clinical services according to Tanzania’s National Guidelines for the Management of HIV. Viral suppression rates are expected to be high, but a small subset of participants will have detectable viral load (≥ 1000 copies/ml) during the study period and will therefore meet health facility criteria for EAC. SOC EAC includes the standard provision of three, once-monthly, 60-minute individual, counselling sessions with a trained counselor on the clinical staff. EAC sessions focus on the meaning of viral loads and supportive, non-judgmental strategies to bolster adherence and visit attendance.

Participants randomized to the intervention arm will receive the same standard HIV care services plus the offer of up to three 22,500 TSH incentives if visit attendance and attendance at each of the three adapted PKC sessions is confirmed using a clinic-operated mHealth system PKC, which is adapted from SOC EAC, is viral suppression “agnostic”, focuses on *potential* barriers to adherence and is intended to address the challenges of long-term HIV care engagement with respect and empathy. It was co-created with experienced ‘HIV counselors’ and mental health professionals/psychologists at the study facilities with input from PLHIV who are currently retained in care. The resulting PKC guide covers many of the components of EAC (e.g., stigma, mental health, coping with HIV, practical tips for success) but also includes motivations/barriers to staying in HIV care, status disclosure, treatment supporter check-ins and long-term health goals and planning. In the three 1:1 sessions, participants will work with the trained health facility counselor to navigate barriers and create a plan to stay in care.

#### Randomization and masking

##### Phase 1 – Out-of-care PLHIV:

The 32 randomly selected facilities will be randomized 1:1 into either the comparison or the cash transfer intervention group (n = 16 health facilities/arm) using a region-stratified, covariate-constrained randomization process (52) to ensure that the arms are balanced on important covariates including: geographic region (Geita, Kagera), facility level (hospital, health center or dispensary), driving distance to a major city (kilometers), proximity to a major road (< 5 kilometers), and log ART caseload (average per quarter from 2021). The 32 health facilities will be randomized 100,000 times. We will select the unique schemes as the randomization space; iterations with an l2 balance score < q = 0.1 will be retained. We will check for validity of the constrained randomization (e.g., no deterministic allocation of clusters into arms) and ensure that there are sufficient constrained randomizations from which to randomly select a randomization scheme among remaining iterations where there was minimal imbalance detected. Due to the nature of the intervention, facility staff will not be masked to intervention assignment. However, other than the facility in-charge and facility Medical Directors, health facility staff will not be informed that there are intervention and comparison facilities in the study, and clinical staff trainings will be conducted separately depending on arm of randomization. Participants will not be told during the consent process that study procedures differ for people living in catchment areas of intervention and comparison facilities.

##### Phase 2 – At-risk, in care PLHIV:

Participants will be randomized in a 1:1 ratio (n = 346 PLHIV/arm, N = 692 total), stratified by site, to comparison or intervention. We will use the *ralloc* function in Stata (53) to create computer-generated random permuted blocks of variable size between 2, 4, 6 and 8, with an equal allocation ratio between the two arms and stratified by study clinic. At the time of enrollment, a member of the study staff will randomize clients to the intervention or comparison arm based on this pre-determined randomization scheme. Due to the nature of the intervention, study participants, study staff, and health facility staff will not be masked to intervention assignment.

#### Assessments and data collection

##### Phase 1 – Out-of-care PLHIV

**Figure 2:**
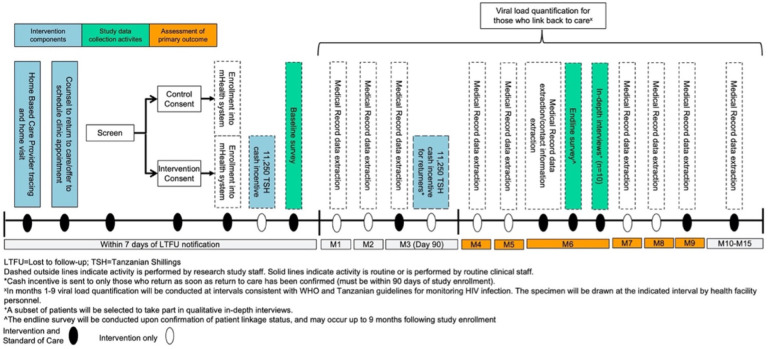
Phase 1 Study Flow Diagram.

Following enrollment HBCs will place a removable sticker on the outside of study participant-held medical record cards to identify clients as participants in the study. Next, study staff will register participants’ information into the study database, including registration of mobile money information (intervention participants only). Upon enrollment, the system will automatically transfer the first half of the incentive (11,250 TSH) via mobile money to those in the intervention arm. The HBC will explain that should they return to HIV care within 90 days of study enrollment, they will receive the second half (11,250 TSH) of the incentive (Fig. 2).

Health facility staff will monitor participants for return to HIV care by identifying the sticker on patient-held medical record cards among clients presenting for HIV care; in addition, study staff will conduct regular medical record data abstractions (Fig. 2) to assess for return to care. If return within 90 days is confirmed, entered clinical visit information will trigger the database to automatically send the second half of the incentive (11,250 TSH) to the participant. All payments will include extra funds for transaction fees, typically <$1. All participant data will be entered into the study Research Electronic Data Capture (REDCap) database, a secure web-based application used for data collection and management in clinical research.

Other study procedures for participants enrolled in phase 1 include:

1. **Baseline and endline survey (n = 640)**: Trained study staff will administer a structured baseline survey via phone to collect participant demographic, socioeconomic, and clinical information. The endline survey will be conducted either over the phone or at the health facility by trained study staff no earlier than 6 months post enrollment and only once linkage status has been confirmed.
2. **Medical record data abstraction**: For participants who re-engage in care, study staff will retrospectively abstract data into study databases, including appointment attendance, HIV viral load, pharmacy dispensing, and current follow-up status.
3. **Viral load quantification**: Viral load quantification will be conducted at intervals consistent with World Health Organization (WHO) and Tanzanian guidelines for monitoring HIV infection after reengagement with care (SOC in all facilities). For those whose standard viral load draw schedule does not include a measurement at 6 months following study enrollment, a research viral load will be drawn.
4. **Primary outcome measurement**: We will assess the primary outcome of viral suppression 6 months after study enrollment. Those not on ART or with virologic failure (≥ 1000 copies/ml), or those who have died will be classified as not having the primary outcome. If there is no evidence of a documented HIV care visit in the participant’s medical record at any facility by 6 months after study enrollment, we will follow tracing procedures to confirm whether the individual is out of care, and if so, he/she will be classified as not in care, and not virally suppressed.

##### Phase 2 – At-risk, in care PLHIV

All participants will be enrolled in a clinic-operated mHealth system and asked to scan their fingerprint to register into the system. Intervention participants will also provide their mobile money information for registration and will complete the first PKC session ideally on the day of enrollment. Participants in both arms will be asked to scan into the mHealth system using their fingerprint or their medical record identification number after attending all clinical visits following enrollment. Those attending PKC and the subset of control participants enrolled in EAC will also scan in after the conclusion of each session. For intervention participants only, receipt of each 22,500 TSH incentive is conditional upon confirmation of visit attendance and completion of each PKC session, which will be sent automatically through the mHealth system. Each payment will include extra funds for transaction fees. All participant data will be entered into the study REDCap database.

Other study procedures for participants enrolled in phase 2 of RKPK are as follows:

1. **Baseline, midline and endline surveys**: Trained study staff will administer a baseline survey on the same day as enrollment, typically at the health facility. Approximately 6 and 12 months following enrollment, trained study staff will administer a structured midline and endline questionnaire, either over the phone or at the health facility.
2. **Viral load quantification**: Viral load quantification will be conducted per SOC procedures by health facility staff and at intervals consistent with WHO and Tanzanian guidelines for monitoring HIV infection. For those whose standard viral load draw schedule does not include a draw at 12 months following study enrollment, a research viral load will be drawn. Six-month viral load (study secondary outcome) data collection will follow a similar procedure.
3. **Enhanced tracing**: At endline only, study staff will use enhanced routine tracing procedures according to national guidelines with additional robust tracing using the same ‘gold-standard’ tracing methods (≥ 3 tracing attempts using multiple methods) to investigate all potentially LTFU clients, confirm ‘silent transfers’ (those who transfer to new facilities without notification of the prior facility) and deaths, and refer clients to health facilities where missing plasma specimens can be collected for viral load quantification.
4. **Primary outcome measurement**: Viral suppression will be assessed 12 months after enrollment. Those not on ART or with virologic failure or those who have died will be classified as not having the primary outcome. Among those with no evidence of an HIV care visit to any facility by 12 months after study enrollment, we will follow tracing procedures to confirm if they are out of care, and if so, he/she will be classified as not in care, and not virally suppressed.

##### Phase 3

Phase 3 will commence after the conclusion of phases 1 and 2 and will include IDIs with PLHIV who participated in phase 1 or 2, clinical staff at phase 1 and 2 health facilities, HBC providers at phase 1 health facilities, and government stakeholders such as Regional or District AIDS Control Coordinators and RMOs. We will also conduct surveys with health facility staff and HBCs at phase 1 and 2 health facilities.

#### Power and sample size

Sample size for phase 1 was calculated to estimate the effectiveness of the incentive strategy for improving the proportion of PLHIV with suppression viral load at 6 months. We used estimates of 6 month viral suppression extrapolated from retention rates in our local studies (33, 37, 42) and viral suppression rates among PLHIV on ART (54) and an intracluster correlation coefficient of 0.01 (55). We estimated that 72% of out-of-care PLHIV will return to care by 6 months after HBC contact alone, and in Tanzania, 92% of PLHIV on ART have viral suppression (54). Thus, we estimate that 66% of PLHIV who are out of care at study initiation and in the comparison arm will have viral suppression at 6 months. With these estimates, we will have 80% power to detect a minimum detectable effect of 11 percentage points as an absolute increase in the percent of PLHIV with viral suppression at 6 months with 320 participants per arm (n = 640 total, 20 PLHIV/facility). This corresponds to 77% with viral suppression in the intervention arm, a clinically meaningful effect size similar to the pilot. Because attrition is part of the primary outcome, we will not inflate the sample size for LTFU.

Phase 2 will include 692 PLHIV. We estimate that 70 percent of PLHIV in the comparison group will achieve viral suppression at 12 months (33, 37, 56). Thus, we will have 80% power with a two-sided type I error of 0.05 to detect a relationship between the intervention and primary outcome if the proportion with viral suppression in the intervention group is at least 80 percent and 294 clients per group are enrolled. Adjusting for 15% LTFU, we will enroll 346 PLHIV per arm (n = 692 overall). Note that since we finalized the study protocol originally conducted our power calculations, the effectiveness of the newest first-line ART regimen containing dolutegravir (DTG) on durable viral suppression has become increasingly evident in several large cohort studies in sub-Saharan Africa. DTG-based regimens achieve higher rates of viral suppression compared to efavirenz-based regimens and are now recommended by WHO as the preferred HIV treatment option in all populations. Thus, we suspect that our original estimate of 70% viral suppression in the control group at 12 months may be low, although estimates of viral suppression among our subgroup of “high-risk” PLHIV on DTG-based regimens are unavailable; repeating our power calculations to account for a variety of possible ranges reveals that we are well powered for a variety of scenarios for what may occur in the control arm. For example, if viral suppression in the control arm were as high as 85% at 12 months, with our current sample size of 692, we retain at least 80% power to detect a 9 percentage-point difference between intervention and control participants. The expected number of participants proposed for phase 3 activities were based on previous studies where we have reached theme saturation emerging with interviews with PLHIV related to study implementation.

#### Statistical analysis

All primary and secondary outcomes are pre-registered and included in the trial statistical analysis plan (Clinicaltrials.gov; Open Science Framework). For phase 1, our primary, intent-to-treat (ITT) analysis of 6-month viral suppression will be a cluster-based permutation test on the individual-level outcome data, which accounts for clustering within the health facility. Multiple imputation will be used for all missing viral load data for those who re-linked to care but do not have a viral load quantified in our pre-specified assessment window. We will also construct a regression model to derive an RD with a 95% CI. For phase 2, the primary ITT analysis of 12 month viral suppression will be examined by study group using a generalized estimating equation that accounts for clustering within facility and adjusted for health facility, and will be expressed as a RD and 95% CI. For both phase 1 and 2 primary analyses, multiple imputation will be used for outcomes with ≥ 5% missing and for covariates if 5% or more records have missing covariates and would therefore be excluded from adjusted analyses. Other trial outcomes are outlined in Table 2.

Phase 3 provider survey data will be summarized using descriptive statistics. Qualitative data analysis will be conducted using Dedoose software and will be based on the CFIR domains which will inform the initial coding framework. Qualitative data analysis will follow an open-coding approach (57, 58) and will be based on research questions and study aims. Concepts will be grouped into themes by CFIR domain and will be summarized in an analytic theme matrix.

## DISCUSSION

Renewed attention to the development of innovative strategies to improve lifelong retention in care among PLHIV is urgently needed to reach the ambitious UNAIDS ‘95-95-95’ targets for ending the AIDS epidemic by 2030. In this type 1 effectiveness-implementation trial, we will evaluate an evidence-based implementation strategy – conditional economic incentives – adapted for two types of PLHIV: those out-of-care and those predicted to be at-risk of disengagement using a novel, machine learning-guided approach.

In keeping with a hybrid effectiveness-implementation study design, the RKPK study aims to collect a blend of effectiveness and implementation outcomes to provide better and more actionable public health information for national decision and policymakers. Our study design considered other key elements that would promote ease of potential future scale-up of these strategies in Tanzania and beyond. First, the cluster randomized design of phase 1 of the study will allow us to understand whether there is heterogeneity of the economic incentive strategy implementation across health facilities and regions, providing key insights into how differential implementation may impact outcomes. Aside from surveys and interviews with a subset of participants, study staff have limited interaction with PLHIV in phase 1. By emulating usual care conditions, we aim to establish if strategy implementation is possible in routine settings and will help us to build the case for inclusion in the national strategy, if effective.

Phase 2 is also highly pragmatic in its utilization of routinely collected demographic, visit, and laboratory data captured in the EMR as predictors in our machine learning model. The proof of concept for this approach was shown to have 72.3% accuracy in predicting risk of disengagement among 178 PLHIV in Tanzania (59) and recently others have had similar success in correctly classifying in care PLHIV at-risk of disengaging in Ethiopia, Mozambique, and South Africa (60, 61). Phase 2 aims to add to this small but growing body of evidence demonstrating that routine data can be effectively and accurately leveraged for use in predictive models to identify those at risk of poor clinical outcomes. We will iterate upon these successes by being the first to test whether this approach can be harnessed to proactively direct limited resources to those who may need it the most, and ultimately improve retention in care above and beyond SOC HIV services.

Our proposed study is innovative in several other ways. Economic incentives, which have been shown to be effective for an array of health outcomes including linkage and retention among ART initiates (23, 24) are a powerful yet understudied tool for improving these same outcomes among out-of-care and at-risk PLHIV. At the study’s end, we will have added to the evidence base demonstrating their use for improving lifelong retention in these two important subpopulations of PLHIV, a key research priority in sub-Saharan Africa broadly (22) and central to Tanzania’s strategy for controlling HIV specifically. In phase 2, we will build upon our experience in prior studies and implement a clinic-run mHealth system to distribute cash transfers for the improvement of patient retention in care (62). By leveraging the widespread use of mobile money services in sub-Saharan Africa (63) with the usage of biometric (fingerprint) scanning we provide a simple and reliable mechanism for cash transfer distribution that would be feasible to implement at scale (62). Finally, our implementation strategy appropriately applies principles from behavioral economics and economic theory to influence care-seeking behaviors. In phase 1, we will examine a primarily behavioral economic intervention: a one-time “nudge” to return to HIV care, designed to help PLHIV to overcome present-biased preferences. In phase 2, we will have evidence demonstrating the impact of a longer incentive strategy designed to both impart habit formation as well as help to overcome structural barriers (e.g., costs) of HIV care. Together, these principles explain the potential of our approach to achieve both short- and long-term impact among out-of-care and at-risk PLHIV.

In order to reach the ‘95-95-95’ goals by 2030, strategies that acknowledge the reality that retention in HIV care is a dynamic, lifelong process that necessitates support at different phases are needed. Findings from the intervention strategies implemented in phases 1 and 2 of this study, which incorporated key principles of implementation science, behavioral economics, and economic theory, will add critical evidence to the scant literature describing the utility of economic incentives for improving re-engagement and retention among the subpopulation of PLHIV who struggle with continuity of care. Results from our mixed methods evaluation will identify key barriers and facilitators to strategy implementation. Together, findings from the three phases of the RKPK study will inform possible adaptation and scale-up of these intervention strategies into the national strategy, which increasingly includes economic support programs and investments in machine learning and data science for health (64–66).

## Figures and Tables

**Figure 3:**
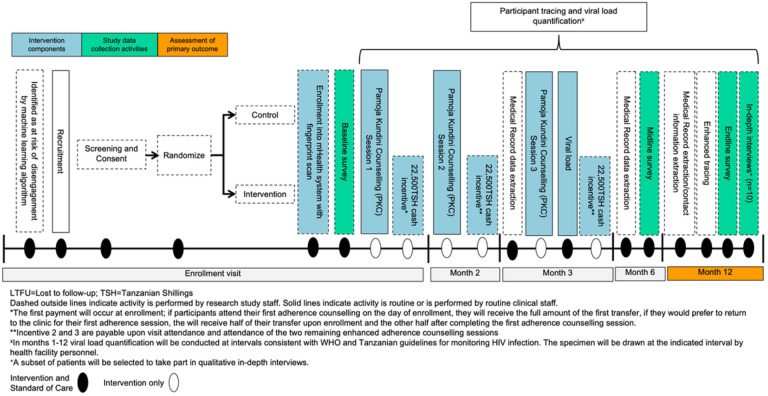
Phase 2 Study Flow Diagram.

**Table 1:** Phase 3 Stakeholder Groups and Data Collection Activities Linked to Domains in the Consolidated Framework for Implementation Research (CFIR, Damschroder, 2009). All data collection activities will occur after conclusion of enrollment for phase 1 or phase 2.

Stakeholder group	Data Collection Approach	CFIR Domain(s)
Health facility staff (directors, head nurses, in-charge physicians, and other facility staff)	Surveys (n = 64)	Inner Setting Intervention characteristics Characteristics of individuals Process
	In depth interviews (~ 20)	
People living with HIV (PLHIV), disengaged from care (Phase 1) or at-risk of disengagement (Phase 2)	In-depth interviews (~ 20)	Intervention characteristics Outer setting
Home-based care providers	In-depth interviews (~ 5)	Characteristics of individuals Intervention characteristics Process
Government stakeholders (District AIDS Control Coordinators, Regional Medical Officers, etc.)	In-depth interviews (~ 5)	Outer setting Process

**Table 2:** Trial secondary outcomes and survey outcomes.

Outcome	Phase 1	Phase 2
*Secondary outcomes*		
Proportion with HIV viral suppression 12 months after enrollment	X	
Proportion with durable HIV viral suppression 12 months after enrollment	X	X
Appointment attendance	X	X
Cumulative incidence of mortality at 12 months after enrollment	X	X
Cumulative incidence of viral suppression at 12 months after enrollment	X	X
Proportion retained in HIV care at 6 months after enrollment	X	X
Proportion retained in HIV care at 12 months after enrollment	X	X
Time to re-linkage to HIV care	X	
Cumulative incidence of re-linkage to HIV care 12 months after enrollment	X	
*Survey outcomes*		
Food security	X	X
Physical, sexual, mental health	X	X
Intimate partner violence/non-partner violence	X	X
Participation in the labor force	X	X
Spending and welfare	X	X
Incentive usage	X	X

## Data Availability

The datasets during and/or analyzed during the current study available from the corresponding author on reasonable request.

## Abbreviations

ART: Antiretroviral Therapy
CFIR: Consolidated Framework for Implementation Research
DTG: dolutegravir
EAC: Enhanced Adherence Counselling
EMR: Electronic Medical Record
HBC: Home-based care
IDI: In-depth interview
ITT: Intent-to-treat
LTFU: Lost to follow-up
PEPFAR: President’s Emergency Plan for AIDS Relief
PKC: Pamoja Kundini Counselling
PLHIV: People living with HIV
RD: Risk difference
REDCap: Research Electronic Data Capture
RKPK: Rudi Kundini, Pamoja Kundini
RMO: Regional Medical Officer
SDT: Self-Determination Theory
SOC: Standard of care
TaSP: Treatment as prevention
TSH: Tanzanian Shilling
UNAIDS: Joint United Nations Programme on HIV/AIDS
USD: United States Dollar
WHO: World Health Organization
